# Fortification of chocolates with high‐value‐added plant‐based substances: Recent trends, current challenges, and future prospects

**DOI:** 10.1002/fsn3.3387

**Published:** 2023-05-17

**Authors:** Amirhossein Abedini, Samira Dakhili, Sara Bazzaz, Saba Kamaladdin Moghaddam, Maryam Mahmoudzadeh, Hashem Andishmand

**Affiliations:** ^1^ Department of Environmental Health Engineering, Food Safety Division, School of Public Health Tehran University of Medical Sciences Tehran Iran; ^2^ Students' Scientific Research Center (SSRC) Tehran University of Medical Sciences Tehran Iran; ^3^ Student Research Committee, Department of Food Science and Technology, National Nutrition and Food Technology Research Institute, Faculty of Nutrition Science and Food Technology Shahid Beheshti University of Medical Sciences Tehran Iran; ^4^ Department of Food Science and Technology, Faculty of Nutrition and Food Science Tabriz University of Medical Sciences Tabriz Iran; ^5^ Drug Applied Research Center Tabriz University of Medical Sciences Tabriz Iran; ^6^ Student Research Committee, Department of Food Science and Technology, Faculty of Nutrition and Food Sciences Tabriz University of Medical Sciences Tabriz Iran; ^7^ Research Center for Pharmaceutical Nanotechnology, Biomedicine Institute Tabriz University of Medical Sciences Tabriz Iran

**Keywords:** functional foods, health effects, nanoencapsulation, phenolic compounds, shelf life

## Abstract

High consumption of delicious foods, such as chocolates, is considered excellent snacks, capable of converting from health‐threatening to great functional foods. The fortification of chocolates with high‐value‐added plant‐based substances might improve their healthful effects, nutritional properties, and shelf life. Chocolate could be an effective carrier for plant‐based substances delivery, and it could be an effective vehicle to treat and reduce the indications of disease, such as obesity, overweight, hypertension, stress, cardiovascular failure, congestive heart failure, and diabetes. Referring to the recent studies in chocolate fortification with high‐value‐added plant‐based substances, it seems that the recent trends are toward its therapeutic effects against noncommunicable diseases. Despite the undeniable functional effects of fortified chocolates, there are some challenges in the fortification way of chocolates. In other words, their functional characteristics, such as rheological and sensory attributes, may undesirably change. It seems that encapsulation techniques, such as spray drying, antisolvent precipitation, nanoemulsification, and liposomal encapsulation, could almost overcome these challenges. Thus, several studies focused on designing and fabricating nanoscale delivery systems with the aim of chocolate fortification, which is discussed.

## Graphical abstract

Chocolate could be an effective carrier for plant‐based substance delivery. The fortification of chocolates with high‐value‐added plant‐based substances might improve their healthful effects, nutritional properties, and shelf life. Despite the undeniable functional effects of fortified chocolates, there are some challenges in the fortification way of chocolates.

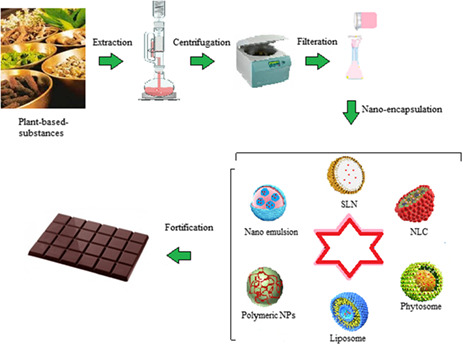

## 1 INTRODUCTION

Chocolate is a functional product in terms of its high flavonoid content and positive effect on human health. Functional foods currently gain prominence in the food market in terms of their ability to provide positive health effects in addition to their traditional nutritional value. On the other hand, chocolate is one of the products which may threaten the health of consumers due to the presence of nondietary compounds in high amounts (Barišić et al., 2019). Chocolate contains antioxidants, anti‐inflammatory agents, and dietary minerals, which may benefit bone health. Other chocolate ingredients, such as cocoa butter, sugar, and methylxanthines, may be harmful to bone health (Seem et al., 2019).

Chocolates, with the ability to activate pleasure centers in the human brain, could be proposed as a great daily snack (Rajeswari, 2020). In recent years, chocolate consumption worldwide has greatly increased among all age groups (Krittanawong et al., 2021). Cocoa is the primary component of chocolate. At the beginning of the 21st century, the annual production of cocoa worldwide was estimated to be around 2 million tons, increasing to around 3 million tons in 2010 and 5 million tons in 2019 (Araújo et al. 2022). Therefore, in terms of this high consumption volume of chocolate, it could be considered not only in the economic sphere but also with proper fortification; it can be turned into a functional product with medicinal effects for disease prevention and treatment.

Using low‐allergic plant‐based substances in formulating chocolates could make them a good transferring vehicle for high‐value‐added bioactive ingredients, which may extend their shelf life (Faccinetto‐Beltrán et al., 2021). Fortified chocolates with high‐value‐added plant‐based substances could be considered rich sources of antioxidants, vitamins, minerals, and fatty acids. The effect of chocolates enriched with apple and extra virgin olive oil on the parameters of cardiovascular disease (Felice et al., 2019) or the effect of oleuropein‐enriched chocolates on diabetes was studied (Del Ben et al., 2020). Hence, consuming the fortified chocolates with high‐value‐added plant‐based substances instead of harmful snacks rich in salt, saturated fatty acids, and sugar can be useful to improve the health of patients with cardiovascular diseases, obesity, overweight, and diabetes (Cicero et al., 2021; Satokari, 2020).

On the other hand, the color and appearance of product are the first parameters which could be affected by fortification (Homayouni Rad et al., 2018). The fortification rate could be varied from 1% to 50%, depending on the food type and consumer acceptance. Consumer acceptance depends not only on the physical or emotional health benefits of fortified food products but also on sensory properties, price, and ease of using products play a crucial role. Rheological or textural properties of chocolates fortified with plant‐based substances could be significantly affected; thus, it provoked industries to use different fortification techniques, such as nanoencapsulation, to overcome induced problems (Chadare et al., 2019).

This review article provides general information on materials that could be used in plain chocolate production, the importance of each production stage, and its role in defects associated with each process. In the following, for the first time, we have paid particular attention to establishing a new connection between the food industry and chocolate's nutritional, health‐promoting, and therapeutic effects fortified with plant substances.

## 2 CHOCOLATE COMPONENTS

Chocolates mostly contain 25%–5% fat. Cocoa nibs have 55% cocoa butter, which forms nearly 30% of the chocolate. Cocoa butter triglycerides are filled with saturated and monounsaturated fatty acids, including 35% oleic, 34% stearic, and 26% palmitic acid. According to its crystal polymorphic form, the chocolate melting point is between 23 and 37°C (Abdul Halim et al., 2019). Cocoa butter forms at least six different crystal forms. From the perspective of appearance and taste, the best chocolate lipid crystal form is V(β2). Due to its high price and rising consumer demand, cocoa butter may be combined with vegetable oil‐based cocoa butter alternatives (CBA). CBA could be classified into three groups, including cocoa butter substitutes (CBS), cocoa butter replacers (CBR), and cocoa butter equivalents (CBE). CBE could be mixed up to 5% with cocoa butter without affecting on physicochemical properties of produced chocolate. CBR should be mixed with cocoa butter so that physicochemical properties are not changed. Hydrogenation should be used on oils, such as soybean oil, rapeseed oil, olive oil, and palm kernel oil, to produce suitable CBR so that they could show possible health problems (Suri & Basu, 2022).

Chocolate contains 50% sugar in the form of sucrose or lactose (in milk chocolate). Fructose or nonsugar bulk sweeteners like sorbitol are used in products for people with diabetes. Due to consumer demand, tooth‐friendly or lower calorie chocolates were produced. Both sugarcane and sugar beets have the same naturally occurring crystalline disaccharide. Sucrose could be converted to inverted sugar using an acidic treatment or the invertase enzyme (Montagna et al., 2019). Sucrose contains more than 40%–50% of solids dispersed in fat and could affect functional properties, such as particle size, sweetness, mouthfeel, and rheological properties of chocolate (Lagast et al., 2018). A 1%–2% change in sugar content affects the price of chocolate and a 5% change affects the flavor (Medina‐Mendoza et al., 2021). As part of cow's milk, lactose is used as crystalline lactose to replace part of the sucrose. Two types of lactose are considered, namely A and B. Form A is produced by formal processes and less sweet and soluble than B. Glucose, called dextrose, is difficult to completely dry because it typically contains some water and can absorb water from the surrounding air. Moisture absorption makes molten chocolate very thick and tends to stick together. Fructose has fewer health problems than sucrose, and because of the lower increase in blood sugar, it could be used to produce chocolate products for people with diabetes (Barišić et al., 2020).

Sugar alcohols are used to produce low‐calorie or sugar‐free products. Different sugar alcohols have varying amounts of calories. However, the average is 2.4 kcal/g. Similar to fructose, they are appropriate for diabetics, but they do not promote tooth rot. Polydextrose is an additional form of sugar comprising glucose units cross‐linked with trace quantities of alcoholic sugar. Polydextrose is a potential prebiotic, producing 1 kcal/g calorie (less than half of the calorie produced by alcoholic sugars) with minor laxative effects (Sözeri Atik et al., 2020). Other components which are usually used in the chocolate formulation include milk powder, soy lecithin, polyglycerol polyricinoleate (PGPR), and vanillin (Selvasekaran & Chidambaram, 2021). Moreover, chocolate is one of the products with a broad diversity, and thus materials should be adjusted based on product type.

## 3 CHOCOLATE PRODUCTION PROCESS AND RELATED REACTIONS

Chocolate production is intricate and involves several stages, such as fermentation, drying, roasting, refining, and conching, which affect the quality of the final product (Table 1). During these stages, chemical reactions for the formation of the ideal flavor and aroma of chocolate occur (Barišić et al., 2019). One of the most important reactions which happen in the roasting process and during which aromatic compounds are created is the Maillard reaction. The Maillard reaction occurs between amino groups from proteins or amino acids and carbonyl groups from reducing sugars (Youssef, 2019). These procedures provide cocoa with its distinctive flavor and aroma. The condensation result of these reactions is converted into 1‐deoxy‐2‐ketosyl via the Amadori rearrangement. Fermentation is less successful at producing amines and aldehydes than roasting. Aldehydes are produced from amino acids in roasted beans by Strecker‐type processes (Tunick & Nasser, 2019).

**TABLE 1:** Effect of different chocolate processing stages on its quality.

Processes	Efficiency	References
Fermentation	Proper fermentation is necessary to generate a unique flavor in the final product The bean is dried out in fermentation, so it cannot be spoiled by germination During fermentation, lots of special chemicals responsible for the creation of flavors in cocoa beans are formed	Assi‐Clair et al. (2019), Beckett (2018), Nurhayati and Apriyanto (2021)
Drying	After fermentation, the beans must be predried and transported to chocolate factories. Any deficiency during this process will encourage mold to grow on the beans. Mold growth limits the product's usability because of its unpleasant appearance and bad taste. The beans can taste very acidic if they are dried too quickly, so it is preferable to dry them at lower temperatures or by intermittent drying	Beckett (2018), Santander Muñoz et al. (2020)
Storage and transport	If the moisture level of beans comes up to 8%, the mold will grow, so they must be stashed in a way that limits water absorption	Barišić et al. (2019), Beckett (2018)
Cleaning, breaking, and winnowing	These processes make the nibs clean (free from any insects and untidiness), fragile, and appropriately dehulled	Barišić et al. (2019), Beckett (2018)
Sterilization	All microorganisms are killed when the nibs or cocoa beans are exposed to high temperatures for an extended time. The method reduces the total plate count (TPC) to <500 CFU/g while also removing all pathogenic bacteria	Beckett (2018), Montagna et al. (2019)
Alkalization	Depending on the alkali, the goal could range from flavor and color adaptation of cocoa liquor to improved dispersibility of cocoa solids in water	Beckett (2018)
Roasting	Many beneficial characteristics of beans, such as flavor, color, and texture, are formed during heating. The roasting process conditions affect the stability of polyphenols in cocoa beans	Ac Pangan (2021), Beckett (2018)
Mixing	Time–temperature combinations in continuous or batch mixers are used for mixing substances during chocolate processing to achieve a consistent formulation	Melo et al. (2020)
Refining	In modern chocolate confectionery, a smooth texture product is so desirable, which was obtained by refining Refiners influence agglomerate degradation and particle size reduction, distribute particles across the contrast level, and cover each with lipid	Ashkezary et al. (2018), Melo et al. (2020)
Conching	Conching is the last step in producing milk or dark chocolate, which affects the viscosity, final texture, and flavor	Melo et al. (2020), Toker et al. (2019)
Tempering, lipid crystallization, and continuous phase character	A properly tempered chocolate would have an appropriate color, good shape, mold contraction, shininess, improved weight regulation, stability, high heat resistance, and a longer shelf life. When chocolate is not properly tempered, type IV crystals form, which quickly transform into form V. Because mirrored light is disoriented by instability and disorganized crystal growth, this transformation affects color	Pirouzian et al. (2020)), Suri and Basu (2022)
Casting and molding	Any changes in the viscosity of the melted chocolate will stop during filling, affecting the size and weight of the finished product. Detempering occurs when the molds are extremely hot, resulting in the product sticking to the mold impressions, poor shine, and blooming. If they are extremely cold, they will lose their shine and stick to the mold, resulting in more air bubbles and markings on the final product	Beckett (2018), Toker et al. (2021)
Cooling	The molds containing melted chocolate are passed through the freezing section after vibration. This mechanized process involves passing the molds through a cooling chamber, reducing the chocolate temperature to around 12–15°C. The chocolate will solidify into bars	Beckett (2018), Sato et al. (2021)
Wrapping/packaging	The foil creates the strongest protection against water vapor and gas transmission. It helps to maintain the chocolate fragrance at a cool temperature. The chosen paper material should be durable, easy to print on, and at a reasonable price. Then, the chocolate is labeled with the batch number, production date, and expiration date by the machine	Beckett (2018), Gunaratne et al. (2019), Kovač et al. (2019)

According to the studies, Maillard reaction is intensified at upper roasting temperatures (135 and 150°C) and produces melanoidins, which are high‐molecular‐weight (HMW) chemicals that give cocoa beans their brown color and distinctive texture. Since cocoa beans have a lower carbohydrate content than coffee beans, it would seem that the formation of HMW melanoidins is more likely to be mediated by lipid oxidation products than by carbohydrates (Tessier, 2021).

During conching, which is the last step of mixing chocolate components and has a great impact on taste, caramelization and Maillard reaction occur. During conching, the amino acid content of chocolate does not change. Its purposes are (1) to remove acetic acid from fermentation and excess moisture and (2) to create favorable changes in viscosity and organoleptic properties (Toker et al., 2019).

## 4 FORTIFIED CHOCOLATES

In recent years, fortified chocolates have gained a great deal of attention due to their nutritional and functional advantages, as well as their potential to suit the demands of health‐conscious individuals who want a better lifestyle. Nutritionists recommended decreasing the consumption of sugary foods to prevent obesity. Fortified chocolates with desirable sensory and taste characteristics could be helpful snacks for obese people (Kaltsa et al., 2021). Furthermore, in terms of unhealthy substances in the chocolate composition and low‐protein content, fortification provides a safer and more nutritious product for food market demands. It positively affects body metabolism at all ages (Kruger et al., 2020).

In previous studies, various foods were considered carriers of particular compounds due to their proper use to treat different diseases. The fortification of chocolate with plant‐based substances has made it a functional product with effective health properties (Faccinetto‐Beltrán et al., 2021). The fortification of chocolate with specific essential components results in a greater nutrient density and sometimes an extended shelf life (Poliński et al., 2021). In recent years, chocolate products were produced from different dried fruits, such as raisins, cranberries, strawberries, apricots, plums, and cherries, rich in easily absorbable compounds, such as minerals, vitamins, and phenolic compounds, which lead to a balanced diet. For instance, it was mentioned that raisins are rich in vitamins, minerals, enough sugar content, and antioxidants like cranberries. Furthermore, strawberries contain not only high amounts of antioxidants such as ellagic acid, quercetin, kaempferol, and flavonoids, which are one of the first disease fighters, but also sufficient amounts of vitamin C, potassium, fiber, and folate. Furthermore, apricots, dried plums, and cherries are good sources of potassium, a wide range of vitamins, and phenolic compounds (Ötleş, 2009).

Milagres et al. (2019) focused on developing a 70% cocoa chocolate containing 8.8% of high‐quality protein, 29.43% of lipid, 57.76% of carbohydrates, 2.30% of fixed mineral residue, and also triterpene acids, such as ursolic and oleanolic acids isolated from *Mansoa hirsuta* DC. Many studies confirmed the imperative role of chocolate polyphenols (flavan‐3‐ols as the major class) in human health. Hence, phenolic concentration augments (1035.45 ± 14.81 mg/100 g of chocolate) and also a significant increase in turmeric dark chocolate phenolic content (1094.03 ± 10.15 mg/100 g of chocolate) were achieved in fortified dark chocolate with Sakura green tea leaves (Martini et al., 2018).

Several studies show that antioxidant capabilities are mostly determined by the basic materials they are derived from. White chocolate's polyphenol content and antioxidant activity are decreased due to the lack of cocoa liquor. This property can be measured by several methods, such as 2,2‐diphenyl‐1‐picrylhydrazyl (DPPH) scavenging activity, 2,2‐azino‐bis‐3‐ethylbenzothiazoline‐6‐sulfonate (ABTS) assay, and reducing power method (Godočiková et al., 2017).

Tea is considered the richest source of bioactive compounds for chocolate fortification. For instance, Lončarević et al. (2019) studied different characteristics of white chocolate with encapsulated green tea extract in amounts of 60, 80, and 100 g/kg, and this fortification not only led to an improvement in total polyphenol content (mg gallic acid equivalents/kg) from 0.41 in white chocolate to 2.73 in the fortified sample with maximal green tea extract, but also during 12 months of storage, its retention was in the range of 62.73%–77.66%. Godočiková et al. (2017) compared the addition of different tea types (green tea Darjeeling, green tea Matcha, and black tea Earl Grey) to white chocolate samples that resulted in more than two times the amount of polyphenolic components enhancement and several‐fold antioxidant activity improvement in white chocolates which were mainly obtained with the addition of green teas, especially in the sample of chocolate flavored with Matcha tea powder.

To increase the overall phenolic content and antioxidant power of white chocolate, Poliński et al. (2022) added ground Matcha green tea (*Camellia sinensis* L.) and moringa (*Moringa oleifera*) leaves at four levels (1%, 2%, 3%, and 4%) during the two stages of chocolate processing (conching and tempering). The results showed that chocolates tempered with powdered Matcha green tea leaves had the highest antioxidant properties compared to others. Furthermore, increasing the plant leaf powders from 1% to 4% increased the antioxidant capacity. It is perceived that the stage of chocolate fortification with active substances could be a significant factor to determine the prohealth properties of chocolate because adding active substances during coprocess may have a negative effect on the polyphenol content and antioxidant capacity of the final product.

Carvalho et al. (2018) showed that adding lyophilized grape and kale could increase the mineral content of milk chocolates with kale extract, as was concluded from the 8.6% of ash enhancement. According to a high‐performance liquid chromatography with a diode‐array detector (HPLC‐DAD), the transfer of phenolic compounds from grape and kale to the final fortified samples improved the level of polyphenolic compounds. Nonetheless, the antiradical activity of milk chocolate could not have been improved in terms of the interaction of the benzene ring and hydroxylic groups of phenolic compounds with the carboxyl or amine groups of proteins, lipids, and carbohydrates. On the other hand, sugar content analysis revealed that lyophilized fruits such as grapes are suitable sugar substitutes in the formulation.

Sun et al. (2021) developed functional dark chocolates fortified with oleogels (12%) obtained from β‐sitosterol in various ratios (γ‐oryzanol to corn oil at a 2:3 ratio, stearic acid to corn oil at a 1:4 ratio, and lecithin to corn oil at a 4:1 [w/w] ratio). The findings of this study show that among the three oleogels, the one containing β‐sitosterol and γ‐oryzanol had the hardest texture, the greatest ability to bind oil, and the strongest heat resistance because of the development of intermolecular and fiber tube structures. Chocolate made with γ‐oryzanol‐based oleogels (cocoa butter and oleogels in a 1:1 ratio) had comparable rheological, crystal structure, texture, and sensory properties to dark chocolate. It appears that the use of oleogels made with bioactive substances that have lower saturated and trans‐fatty acid levels in the chocolate industry could play a significant role in enhancing consumer health.

Spirulina, a microalgae with great protein (nearly 70% of the biomass) and amino acid content, is an innovative and promising substance for novel chocolate formulation. Hence, Şahin‐Cebeci (2019) showed that incorporating 2% (w/w) Spirulina in homemade chocolate had nearly four times the protein content of commercial baby foods, and it could meet the required daily intake of essential amino acids, histidine, and arginine for infants and children. Raja et al. (2021) increased the amount of physiologically active substances in chocolate by including *Centella asiatica*, *Abelmoschus esculentus*, and *Psidium guajava* in the formula compared to conventional chocolates, the optimized product contained more calcium (67.8 mg), iron (2.34 mg), and sodium (355 mg) per 100 g (13 mL of *C. asiatica*, 13 mL of *A. esculentus*, and 24 mL of *P. guajava*). Moreover, the improved product shows antibacterial activity against the bacteria *Lactobacillus acidophilus* and *Streptococcus mutans*, which cause tooth decay. A 30‐day shelf life for the product was discovered. It seems that their novel product synergizes phytomedicine and chocolate.

Based on Kumari et al. (2021), as a source of phenolic, flavonoid, and carotenoid compounds, butter fruit milkshake powder, a nutritious blend of avocado pulp and dairy ingredients with high nutritional and antioxidant activity, was added to functional chocolate at a level of 30%. They said that the formulation of the chocolate now had more of the minerals Ca, Mg, Fe, Zn, and Mn. Furthermore, it was more stable than the control chocolate and was well tolerated by customers for storage times of up to 60 days. This study showed that chocolate supplemented with BFMS powder improved nutritional value, was reasonably priced, and had acceptable sensory qualities. Studies showed that the fortification of chocolates with different plant‐based substances mainly results in higher phenolic content, and consequently, improved antioxidant activity, which is an essential factor, especially in white chocolates which fundamentally contain lower amounts of phenolic components. Besides, these fortified chocolates with higher amounts of protein and minerals can slightly compensate for the deficit of these nutrients.

## 5 NANOENCAPSULATION OF ACTIVE SUBSTANCES

**FIGURE 1:**
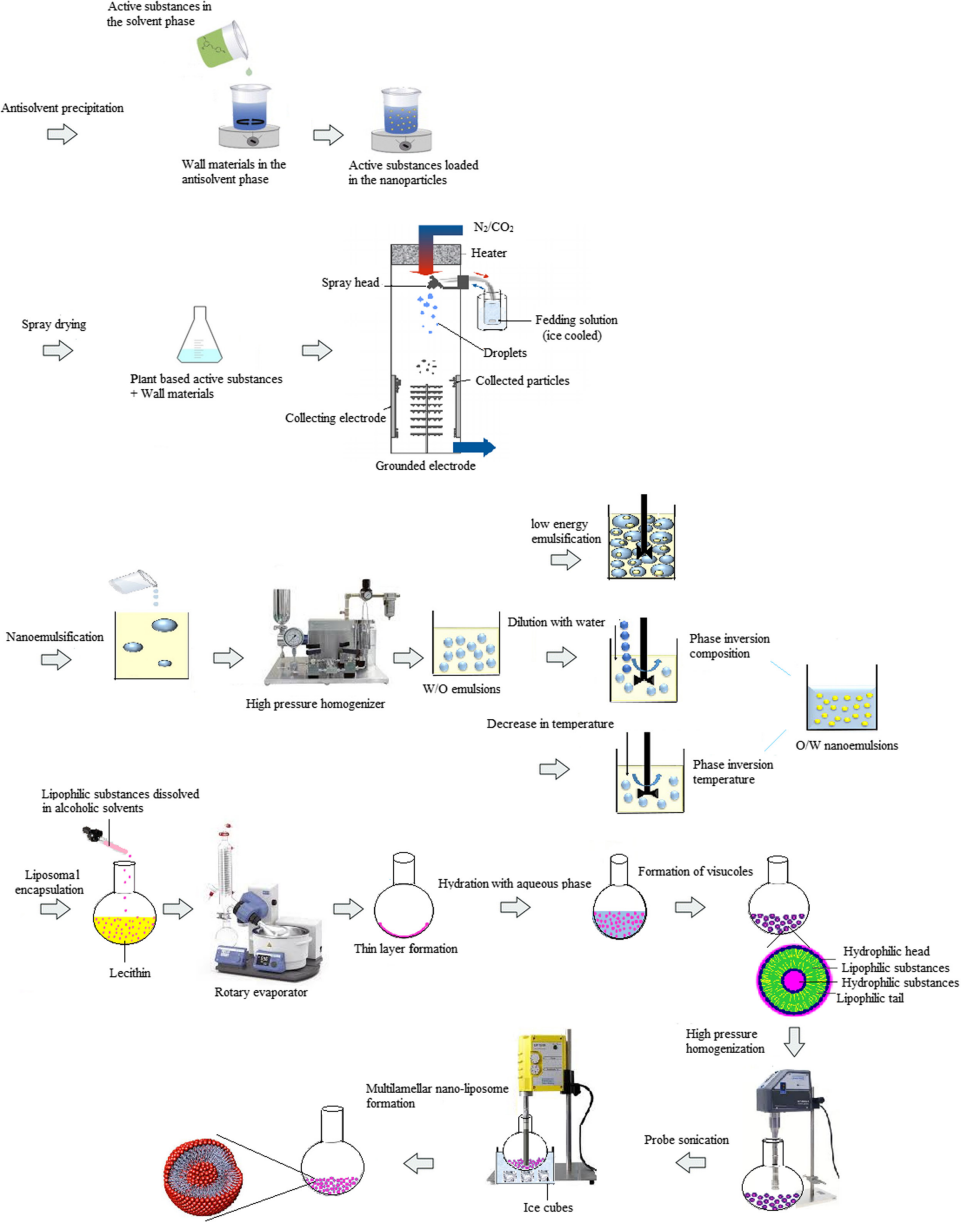
Preparation steps of nanoscale delivery systems for encapsulating plant bioactive agents.

The stability, bioactivity, and bioavailability of active substances are essential to develop fortified foods (Andishmand et al., 2016). Food fortification is challenging because of the incompatibility of the fortifier with the food matrix, storage stability, and the possibility of changes in sensory characteristics (Faccinetto‐Beltrán et al., 2021). Therefore, different delivery techniques rather than the direct addition of active substances were developed to overcome the mentioned problems and make them industrially applicable (Andishmand et al., 2017). Techniques used to nanoencapsulate plant‐based substances can be classified as (1) physical methods (such as spray drying, antisolvent precipitation, freeze‐drying, extrusion, fluid bed coating, and supercritical fluids); (2) chemical methods (such as polymerization and inclusion complexes); (3) physicochemical methods (such as emulsification, liposomal encapsulation, complex coagulation, and ionic gelation); and (4) electrohydrodynamic methods (such as electrospinning and electrospraying) (Hosseini & Jafari, 2020). Some of these methods were used for chocolate fortification, which is discussed next (Figure 1).

### 5.1 Spray drying

Spray drying is the most common method of encapsulating food substances. This method is economical and widely used to flavor compounds. The carrier or wall, such as maltodextrin or another gum, is hydrated in this process, and the ingredients to be encapsulated are added and combined in a certain ratio. The mixture is then injected into the system to create a small droplet (Piñón‐Balderrama et al., 2020). When the droplets are exposed to hot air, they quickly lose their moisture, and spray‐dried particles are assembled in the product chamber. As a result of the high speed (a few seconds) of the drying process, the product suffers the least loss of biofunctional properties (Assadpour & Jafari, 2019). Working with highly viscous feeds is possible via preheating, particle size, shape, and morphology control, and the design of particles with controlled release properties (Cetinkaya et al., 2021), spray‐drying process of several types of juices and concentrates (Sarabandi et al., 2018), herbal extracts (Sarabandi et al., 2019), and vitamins (Terracina et al., 2022) was studied.

Dean et al. (2016) studied chocolate fortification with peanut skin extracts to evaluate consumer acceptance. Maltodextrin feeding into a spray dryer reduced the phenolic component extracts' bitterness by encapsulation. At the lowest inclusion level (0.01%), 24% of the untrained panelists were able to identify the threshold for the inclusion of encapsulated chocolate infused with peanut skin extract. The best threshold (BET) of each panelist was calculated for this purpose using the American Society for Testing and Materials (ASTM) standard method. The geometric mean of the individual's BET was then used to calculate group BET. To assess the level of chocolate flavor, sweetness, bitterness, and creaminess, a 5‐point “just‐about‐right” scale was used. To determine how sweetness, creaminess, and bitterness affected mean chocolate liking, penalty analysis was used. Maltodextrin had no negative effect on chocolate texture, according to the results. Furthermore, penalty results revealed that excessive bitterness or sweetness significantly increased the overall mean liking of the dosed product.

Ekantari et al. (2019) studied the stability of fortified chocolate by adding nanocapsules of *Spirulina platensis* carotenoid to enhance premium nutrition. By encapsulating substances with various coatings using the spray drying method, nanocapsules were produced. The parameters affecting blooming development, color fading over time in storage, melt point, and consumer approval were examined. Fortification improved the color and look of chocolate, whereas nonfortified chocolate soon produced fat blooms. Adding *S. platensis* nanocapsules not only did not change the melting point of chocolate, but also increased the chocolate shelf life by 1.5 times longer (Ekantari et al., 2019).

Lončarević et al. (2019) encapsulated green tea extract using a spray drying technique to increase the antioxidant capacity (AC) and total polyphenolic (TP) content of white chocolate. Based on used green tea extract content, fortified chocolate had higher viscosity, volume‐weighted mean, antioxidant, and total polyphenol content. Finally, green tea extract encapsulated in maltodextrin increased chocolate shelf life by at least 12 months.

Lončarević et al. (2018) used encapsulated blackberry juice in different concentrations of 60, 80, and 100 g/kg in another study. Blackberry juice was encapsulated with maltodextrin using the spray drying method. To create fortified chocolate, encapsulated material was combined with additional ingredients such as cocoa butter, sunflower lecithin, sugar, and vanilla powder. Adding encapsulated blackberry juice increased the caisson viscosity of chocolate in terms of the increase in the specific surface of solid particles. Finally, encapsulated blackberry juice created a new material with a pleasant taste and color.

### 5.2 Antisolvent precipitation

Another fortification method is antisolvent precipitation in which ultrafine enriching agents are prepared. The basic principle is that the enriching bioactive agents are dissolved in a solvent; the solvent solution is then mixed with an antisolvent. Finally, precipitates are produced, creating conditions that produce spherical particles with smooth surfaces in nano or micro dimensions and a narrow particle size distribution (de Boer et al., 2019). This technique is significantly used in the pharmaceutical industry, but some researchers investigated it for food fortification. Rather than water‐soluble compounds, this method is best suited for encapsulating lipophilic bioactive agents. When nanocapsules are prepared, high loading efficiencies (owing to oil‐based central cavities of the nanocapsules) are reported on lipophilic substances. Using potentially toxic and environmentally unfriendly organic solvents to dissolve the flavonoids is one of the major drawbacks of the traditional antisolvent precipitation method. Thus, in recent years, producing the nanoparticles was developed using supercritical CO_2_ (ScCO_2_), which is nontoxic and easily separated from the products (Kaga et al., 2018).

Muhammad et al. (2018) studied the fortification of chocolate with engineered cinnamon nanoparticles to improve the polyphenol content and enhance antioxidant activities. According to earlier research, adding cinnamon directly to chocolate reduces its melting profile, rheological behavior, textural qualities, and sensory aspects while also acting as an anticancer and health‐promoting fortifying spice. Hence, this study aimed to focus on the antisolvent precipitation technique as a promising method to deliver cinnamon extract. To that end, antisolvent precipitation of colloidal nanoparticles in cinnamon extract (LCNP‐CE) was used, followed by freeze‐drying. The antisolvent precipitation method produced LCNP‐CE, which was used to fortify white and milk chocolates from 0% to 2% w/w. Results showed fortification with LCNP‐CE improved the polyphenol content and antioxidant activities of white and milk chocolate. Despite minor changes in quality factors (hardness, flow behavior, and color), the final product was within an acceptable range.

### 5.3 Nanoemulsification

Nanosized emulsions were applied as one of the delivery methods in fortification. The size of this colloidal system varies from 10 to 1000 nm. They have several advantages, including increased physical stability, large surface area, ability to use in various formulations, and improved taste sense. Besides, nanoemulsification has a wide range of applications in food and nutrition, biology, and pharmacology, particularly in high‐efficiency encapsulation and directed delivery of substances (Andishmand et al., 2023; Islam et al., 2022). Different materials may be used to create various colloidal delivery systems based on emulsification with various characteristics and architectures. As a result, it seems to be appropriate for hydrophilic and lipophilic chemicals in various food matrices. However, the costly formulation procedures and the need for a special tool and instability mechanism are the drawbacks of this method (McClements & Jafari, 2018).

Hence, Ekantari et al. (2019) studied the effects of milk and dark chocolate fortified with carotenoid from *S. platensis* by nanoencapsulation. They used this method to cover fishy odor induced during direct biomass addition. The mixture of Arabic gum and whey protein concentrate was used to encapsulate *S. platensis* carotenoids by nanoemulsification. This research studied the nanoemulsification effect on dark and milk chocolate flavors. Gas chromatography–mass spectrometry (GC–MS) was used to detect active aroma in dark, milk, and fortified chocolate. Results showed dark chocolate was dominated by a strong acidic aroma, whereas milk chocolate was dominated by a creamy, cheesy, and sweet aroma. Fortified chocolate had a lower acidic aroma, but higher pyrazines and alcohols. The nanoencapsulation technique promoted sweet and chocolate flavors, but lowered bitter and sour flavors.

### 5.4 Liposomal encapsulation

Liposomes are made up of an aqueous phase surrounded by a phospholipid‐based membrane. Liposomes spontaneously arise when phospholipids are distributed in aqueous fluids (Yousefi et al., 2023). Materials might be enclosed in the aqueous or lipid portion depending on how soluble they are. Water‐soluble phenolic chemicals are confined in the water core, while water‐insoluble phenolic compounds collect in the lipid section (Andishmand et al., 2023). Liposomes are created using various organic solvent‐based methods, including ethanol injection, film hydration, and reverse‐phase evaporation (Subramani & Ganapathyswamy, 2020). The smaller nanoparticle sizes, durable phenolic compounds during stomach conditions, and sustaining the antioxidant activity of peptides are reported as the advantages of this method. Other advantages include using natural substances; the simplicity of synthesis; acceptable encapsulation efficiency; high biocompatibility and biodegradability; and the entrapment of water‐soluble, lipid‐soluble, and amphiphilic materials. However, the drawbacks of liposomes are reported to be organic solvent residue, low physical and chemical stabilities, leakage and fusion of encapsulated ingredients/molecules, difficulty in scaling up, and short half‐life (Ajeeshkumar et al., 2021; Hosseini & Jafari, 2020; Ramezanzade et al., 2017). Gültekin‐Özgüven et al. (2016) studied the fortification of dark chocolate with spray‐dried black mulberry waste extract encapsulated in a chitosan‐coated liposome. Dark chocolate production waste contains high anthocyanin content that possesses instability against increased pH and temperature. Cationic chitosan was deposited to coat primarily produced liposomes by the layer‐by‐layer procedure. Results indicated higher stability of chitosan‐coated liposomal powders during increased temperature and pH. The highest fortification efficiency of chocolate with encapsulated anthocyanin was 76.8% and varied based on conching temperature and pH.

Finally, in addition to stability against processing conditions, liposomal encapsulation could extend the oxidative and storage stability of encapsulated substances; thus, it could be a great suggestion for use in plant‐fortified chocolate production, which is an excellent source of polyphenolic compounds.

### 5.5 Fate of nanodelivery systems in the human body

Delivery systems in the nanoscale may have diverse biological fates in the human body, such as levels of absorption, excretion, distribution, and metabolism (Katouzian & Jafari, 2016). The biological fate of nanocarriers depends on their physicochemical properties (e.g., dimensions, composition, method of preparation, structure, surface properties, and physical state) as well as the changes they undergo while passing through the gastrointestinal tract (GIT). For example, the biological fate of lipid nanocarriers varies depending on whether they are directly absorbed or digested by the human body (Jahangirian et al., 2017). Smaller indigestible nanoparticles accumulate in organs more rapidly compared to the larger sizes. In addition, nondigestible metallic (Ag and Au) and inorganic (TiO_2_ and SiO_2_) nanoparticles have been reported to pass through the layers of epithelial cells by different routes such as transcellular, paracellular, or persorption (Abdellatif et al., 2021). Also, inorganic nanoparticles composed of carbonate, phosphate, and calcium may lead to ectopic calcification and kidney stones. In addition, mineral particles may play a role in immune tolerance against food antigens and gut microbiota (Gangadoo et al., 2021). Nanocarriers may be accumulated, digested, or transported into the systemic circulation after absorption in the epithelial cells through the lymphatic or blood system. Nanoparticles may be transported through the human body following accumulation, metabolization, or excretion in certain tissues after exiting epithelial cells (Zhang et al., 2020). However, an in‐depth investigation is desired to reveal the direct absorption fate of indigestible and digestible nanoparticles containing plant‐based substances in humans.

## 6 RECENT TRENDS IN FORTIFIED CHOCOLATES

Functional foods could be used as a nonprescription therapeutic aid and so, despite meeting nutritional demands, their enrichment could promote healthy effects. In previous studies, various foods were considered carriers of particular compounds in terms of their ease of use to cure diseases (Costa et al., 2020). The consumption of fortified chocolates with plant‐based substances, such as polyphenols (phenolic acids, flavonoids, stilbenes, phenolic alcohols, and lignins), was evaluated to treat diseases such as cardiovascular disease, diabetes, hypertension, obesity, improved endothelial cell function, stress, and improved mental function, some of which are presented in Table 2.

**TABLE 2:** Therapeutic effects of fortified chocolates.

Active substance(s)	Study type	Received dose	Inclusion criteria	Exclusion criteria	Main result(s)	References
Apple and extra virgin olive oil	Randomized, crossover	40 g dark chocolate with 10% EVOO or 2.5% dry apples	1. Smoking 2. Dyslipidemia 3. Hypertension 4. Overweight and family history of cardiovascular disease 5. Aged 25–65 years	1. Eating disorders 2. No history of cardiovascular disease, diabetes mellitus, sleep apnea, inflammatory gastrointestinal tract disease, pregnancy 3. Use of anti‐inflammatory medication 4. Restricted diets by choice (i.e., vegan, carbohydrate‐restricted) 5. A known allergy to cocoa or chocolate	Significantly increases the amount of EPC	Felice et al. (2019)
Flavanol	Double‐blind	40 g chocolate containing catechin and epicatechin at concentrations of 0.27 and 0.9 mg/g, respectively	Congestive heart failure	1. Decompensated heart failure 2. Unstable angina pectoris 3. Smoking 4. Creatinine >200 mmol/L 5. Alanine aminotransferase or aspartate aminotransferase >150 IU 6. Diabetes mellitus 7. Obesity (body mass index 30 kg/m^2^) 8. Known allergies to compounds of study chocolate or placebo history of gastric ulcer or bleeding 9. Venous thrombosis or pulmonary embolism 10. History of infectious disease or systemic inflammatory diseases 11. Malignant tumors	Improves endothelial function and increases the concentration of plasma epicatechin in healthy adults	Flammer et al. (2007)
Flavonoid	Double‐blind	Chocolate (213 mg procyanidins, 46 mg epicatechin)	Healthy volunteer	1. Cardiovascular disease 2. Diabetes 3. Hyperlipidemia 4. Thyroid disorders 5. Smoking 6. Vegetarianism 7. Extreme physical activity	Beneficial effects on vascular function in patients with CHF were seen	Engler et al. (2004)
Polyphenol	Double‐blind	45 g of chocolate containing 16.6 mg of epicatechins	Type 2 diabetes	HbA1c >9.0%, treatment with insulin, use of steroids, any change in use of medication during the test interval	Increasing HDL, beneficial effects in improving parameters such as inflammatory markers, insulin resistance, and glycemic indices were seen	Mellor et al. (2010)
Polyphenol	Placebo‐controlled double‐blind	25 g dark and white chocolate with 450 mg flavonoids	Conducted on 35–70 years old patients with type 2 diabetes and hypertension	Insulin consumption	It showed a healing effect on inflammatory parameters, cholesterol levels, and lowered blood pressure	Rostami et al. (2015)
Oleuropein	Randomized, single‐blind, crossover	40 g oleuropein‐enriched chocolate by EVOO (4 mg% oleuropein)	Diabetic patients	1. Chronic or acute liver disease 2. Serious renal disorders 3. Treatment with any vitamin, antioxidant supplements, or insulin in the month preceding	There was no change in the glycemic index or a very slight increase due to incretin reactions	Del Ben et al. (2020)
Polyphenol	Single‐blind	25 g of chocolate containing 500 mg of flavonoids	1. Healthy men and women 2. 18 years and older 3. Nonsmokers 4. Do not use dietary supplements and antioxidants 5. No history and no regular medication for heart disease, high blood pressure, liver or kidney disease, high cholesterol, autoimmune disease, cancer, psychiatric disorders, or diabetes 6. No history of lung, thyroid, neuromuscular, or neuroleptic disease, and no regular medication 7. Not pregnant or breastfeeding 8. No food allergies or food intolerances	–	A decrease in salivary cortisol levels was seen	Tsang et al. (2019)
Polyphenol	Single‐blind randomized placebo‐controlled	20 g of *Mansoa hirsuta* DC containing 500 mg polyphenols	Healthy and nonsmoking (BMI 18.5–24.99 kg/m^2^) people with no history of diabetes, hypertension, or cardiovascular disease	1. Taking dietary supplements, BP or cholesterol‐lowering drugs 2. Taking soy and nut allergies 3. Smokers	Beneficial effect on reducing BMI	Almoosawi et al. (2012)
Polyphenol	Randomized parallel trial	20 g daily chocolate consumption containing 500 mg total polyphenols (400 mg flavanols)	1. Adults with no history of hypertension, type 2 diabetes 2. Participants with BMI between 18.5 and 24.9 kg/m^2^ and BMI between 25 and 34.9 kg/m^2^ 3. Males and females 4. Age: 18–65 years	1. History of CVD, hypertension, diabetes 2. Intake of medications that affect insulin, glucose, lipids 3. Dietary supplements containing high doses of antioxidants (only low amounts of multivitamins were considered acceptable doses) 4. Current smoking and heavy alcohol	Polyphenols had a small effect on BMI and glucose levels and modulated negative effect of fat	Farhat et al. (2015)
Polyphenol	Randomized, single‐blind, crossover	20 g *Mansoa hirsuta* DC with 500 and 1000 mg polyphenols	1. Healthy nonsmoker volunteers 2. Aged 19–50 years and BMI >25 kg/m^2^ 3. No history of diabetes, hypertension, or CVD	1. Smokers 2. People were taking dietary supplements, BP or cholesterol‐lowering drugs, soya, and nut‐allergic people	Decrease in fasting blood glucose levels, systolic BP (SBP), diastolic BP (DBP), and BMI	Almoosawi et al. (2010)
Polyphenol	–	50 g of chocolate with 2135 mg polyphenols	18 and 60 years old people with BMI from 25.0 to 34.9 kg/m^2^	1. Use of antioxidant and dietary supplements 2. Use of any known medication to interfere with body weight, blood pressure 3. Individuals with eating disorders, major depression, or a medical history of drug addiction	Improve the function of endothelial cells	Nogueira Lde et al. (2012)

Abbreviations: CHF, congestive heart failure; CVD, cardiovascular disease; EPCs, endothelial progenitor cells; EVOO, extra virgin olive oil; HDL, high‐density lipoprotein.

Lee et al. (2019) examined the effect of chocolate consumption on hearing loss and tinnitus in middle‐aged people. The results of this study showed that the rate of hearing loss (unilateral or bilateral) was significantly lower in those who consumed chocolate (*p* < .001). Furthermore, the frequency of chocolate consumption is inversely correlated with the severity of the hearing loss. They hypothesized that chocolate because of having cocoa exerts antioxidant and anti‐inflammatory properties that improve neurodegenerative diseases.

Felice et al. (2019) examined the effect of chocolate fortified with apples and extra virgin olive oil (EVOO) on the risk of cardiovascular disease. They used endothelial progenitor cells (EPCs) as a marker in the development of cardiovascular disease. A solid dark chocolate bar, containing 10% EVOO and 2.5% apples, was consumed at a dose of 40 g/day. Participants were 30 subjects with cardiovascular disease, and their urine samples and anthropometric data were collected within 4 weeks. Results showed that daily consumption of fortified chocolate significantly increases the amount of EPC. Higher EPC levels were reported to indicate better endothelial cell function, which could decrease the risk of coronary disease.

Leyva‐Soto et al. (2018) evaluated the effects of daily consumption of flavonoid‐rich chocolates on cellular genotoxicity and biochemical parameters of glucose and lipid metabolism of 84 young volunteers. The treatments included the daily intake of either 2 g of dark chocolate having 70% cocoa or 2 g of milk chocolate for 6 months. The results indicated that dark chocolate significantly prohibited DNA damage and enhanced the nucleus integrity of buccal epithelial cells. This effect could be related to the antioxidant capacity of dark chocolate that decreased cellular stress. Biochemical parameters (LDL‐cholesterol level in blood, triglycerides, and total cholesterol) and anthropometrical parameters (waist circumference) were bettered after 6 months of daily intake of dark chocolate.

Psychological stress and chronic stress are essential contributing factors in the increased incidence of diseases, such as obesity, cardiovascular disease, and diabetes (Florez‐Mendez et al., 2019). Thus, Tsang et al. (2019) studied the health benefits of polyphenol‐fortified chocolate to treat salivary glucocorticoid (GC), cortisol, and cortisone. Stress parameters were evaluated in 26 people working in health and social centers. As shown in Table 2, participants consumed 25 g of chocolate containing 500 mg of flavonoids per day. Results indicated positive changes in the inhibition of 11‐HSD (11‐hydroxysteroid dehydrogenase) type 1 activity, leading to a slight decrease in salivary cortisol levels. Flavonoids can reduce enzymes involved in increased cortisol, including 11‐HSD.

Despite the therapeutic effects observed from the consumption of fortified chocolates, it is necessary to consider researchers' concerns about long‐term or excessive chocolate intake or when no effective concentration of plant‐based substances was used to make chocolate (Halib et al., 2020).

## 7 CURRENT CHALLENGES IN FORTIFIED CHOCOLATES

Nowadays, the development of functional foods like fortified chocolate, in terms of its matrix as a promising carrier for various wholesome compounds, was considered a new era for novel food products which benefit customers concerned about their health. Hence, several medicinal plants and biomolecules from microalgae are often used to achieve prospective healthfulness. However, consumers' assessment of fortified products' quality and sensory characteristics play an important role to accept these products (Ekantari et al., 2019).

The fortification of chocolate with natural products could affect the rheological proprieties that the Casson model could best describe. Based on this model, chocolate's yield stress and plastic viscosity as a non‐Newtonian fluid could be determined for quality and sensorial controls. The formulation's processing conditions and the type of substances could affect yield stress and plastic viscosity (Cahyani et al., 2019). While in some studies, fortification resulted in higher Casson viscosity, in others there was no variation of this parameter, and the interaction of added substances with other constituents like free fat is fundamental to study this character. For instance, yield value and viscosity are reduced by adding fat content, but moisture values >3% can increase initial tension and viscosity (Godočiková et al., 2017).

Moreover, the distribution of particles in chocolate and their interactions is inversely related to its mechanical properties. The most desirable interval is 15–30 μM as bigger or smaller particles may interfere with its palatability. Regarding the bigger particles of fortification substances, a high polydispersity index can be seen in many cases. Furthermore, appropriate melting behavior as another aspect of rheological and textural properties ranges from 32 to 34°C, which is necessary for proper organoleptic perception (Tolve et al., 2018).

Sensory evaluation is a measurement that can be considered quantitative or qualitative indices that provide the understanding of consumers' satisfaction in terms of appearance, color, melt‐in‐mouth, texture, unique flavor–taste, and odor in produced chocolates. Findings from previous research proved that the palatability of these products mainly relies on the degree of their bitterness and mouthfeel. In general, if the correct dose of fortification was chosen, the consumers preferred both plain and fortified samples at close levels (Razavizadeh & Tabrizi, 2021). Moreover, these attributes of fortified chocolate can be affected by various factors, including fermentation and roasting processes and applied flavoring agents. For example, free amino acid and polyphenol compounds may cause significant changes in the formation of chocolate flavor (Kaltsa et al., 2021). Since color is responsible for consumers' first impression, its evaluation via visualization techniques, colorimetric or spectrophotometric methods, is required. Tannins, made up of epicatechin molecules, are the main class of chemicals found in cocoa that are liable for the development of brown color. Furthermore, further factors, such as the production process of formulated substances and the storage time, could determine the color characteristics. There was no significant difference between plain and fortified chocolates in some cases, while in some studies, the latter group looked brighter based on the substances of the added material (Ekantari et al., 2019). In the following, we summarize the functional properties of different fortified chocolates with plant‐based substances (Table 3).

**TABLE 3:** Functional, sensory, and antioxidant properties of fortified chocolate with plant‐based substances.

Chocolate type	Fortified substance(s)	Form of fortification	Rheological properties	Sensory properties	Color	Antioxidant properties	References
Milk chocolate	Lyophilized kale and grape	Free	While plain milk chocolate showed the minimum particle size and higher viscosity, fortified chocolate with grape had a larger particle size and lower viscosity Studies on texture indicated that chocolate hardness is modified substantially with the addition of grapes. Also, the rheological analysis pointed out that the milk chocolate with no treatment had the highest Casson viscosity	Sensory analysis revealed that 47.4% of the panelists expressed approval for “crunchy texture”; however, 52.6% did not like the product for this proof A purchase intention of 61.1% was demonstrated for chocolate with kale, 66.6% for plain milk chocolate, and 64.2% for chocolate with grapes	–	Studies showed that all samples had a high radical scavenging profile. However, the results revealed that there was no augment of antiradical capacity with the fortification, due to the interactions between proteins and polyphenolics	Carvalho et al. (2018)
Dark chocolate	Phytosterols esters (PS) (2.2 g)	Free	Hardness decreased in all samples from initiation (7.3 2.1 N) until 90 days of storage (4.4 1.6 N). After this time, a hardness enhancement (7.7 2.2 N) was seen	The sensory acceptability was not influenced by the color and texture variations. Scores above 6.0 for all samples mean that dark chocolate was acceptable at a good level	Samples saved the color stability until 90 days of storage (L* 27.3 ± 1.5, a* 5.6 ± 0.4, and b* 4.4. ± 0.7). After this period, all samples became lighter (L* 31.8 ± 2.6) and more red‐yellow (a* 6.1 ± 1.1 and b* 6.6 ± 0.8)	The results showed that this study's applied antioxidants at that dosage (0.90 mg/100 g) could not decrease oxidation	Botelho et al. (2014)
White chocolate	*Cinnamomum burmannii* Blume essential oil (0.1% w/w)	Free	The presence of cinnamon essential oil slowly raised the Casson yield stress and the thixotropy, but no changes were observed in the Casson viscosity of white chocolate. Both control and fortified samples had the highest melting rate of 32–34°C	Fortification with 0.1% (w/w) of cinnamon essential oil led to an acceptable flavor perception for consumers, but an unacceptable “too spicy” flavor was perceived at a higher level. Consumers' color differences were not detectable as they were comparatively of similar chroma value	No significant change in the color properties of white chocolate, particularly in the L* and b* values, could be investigated. A slight alteration in the whiteness index was observed due to the difference seen in the a* value	The fortification of chocolate with 0.1% (w/w) of cinnamon essential oil improved the antioxidant activity of the white chocolate more than twofold	Muhammad et al. (2020)
Dark chocolate	Wheat germ (WG) (6–15% w/w)	Free	The addition of ground WG to dark chocolate negatively affected particle size compared to intact WG. Consequently, the recommended optimal fortification level was 10% (w/w)	The appearance and color properties between all treatments were evaluated as “like very much” Fortification with 10% WG in the control dark sample resulted in decreased sensory properties and overall acceptability attributes from “like very much” to “like moderately”	There was no difference between the colors of all treatments	–	Al‐Marazeeq (2018)
Milk and dark chocolate	Nanocapsules carotenoid of *Spirulina platensis* (0.372%)	Encapsulated	No differences in the texture were seen between chocolate with and without fortification by Spirulina	Sensory analysis revealed that the fortification of dark chocolate with nanocapsules improved panelist preference. There were no differences in the chocolate aroma profile between the fortified sample and chocolate brown without Spirulina, but the intensity differed The genuine taste of chocolate could be detected in both dark and milk samples, with a bitter taste	Fortified samples demonstrated a lower *L* value that made the sample darker, but there was no difference between the plain milk chocolate and those with added Spirulina. Besides, it was concluded that the longer storage time increased the *L* value. Nevertheless, no changes in the color parameters of a* and b* in dark and milk chocolate were detected	–	Ekantari et al. (2019)
Milk chocolate	Peanut skin extracts	Free	–	Sensory analysis revealed that the fortified product and the control milk samples were similar at the same level Moreover, texture liking was the same for the plain and dosed samples (7.1)	–	The fortification at a dosage below the best estimate threshold (0.8%) significantly improved the chemical antioxidant capacity of the milk chocolate	Dean et al. (2016)
White chocolate	Blackberry juice encapsulate (60, 80, and 100 g/kg)	Encapsulated	Casson viscosity mass climbed from 0.63 Pas in white chocolate to 1.22 Pas for the fortified sample with a maximum dose of fortification Simultaneously, particle size distribution increased in fortified samples regarding the added concentrations. It was concluded that samples fortified with 100 g/kg (WE10) of encapsulates substantially had a higher hardness value than white and enriched samples with 60 g/kg (WE6) of encapsulates	Panelists understood that fortification decreased the melting time. Fortified samples showed higher smoothness scores than white chocolate; however, there was no significant difference among them On the other hand, higher encapsulates in this fortification noticeably improved the intensity of the fruity taste, even though sweetness declined	Fortification of white chocolate with encapsulating (E) decreased the values of L* in enriched samples, where WE6 showed a significantly higher value of L* than E, WE8, and WE10, which did not differ significantly from each other Nevertheless, there was no significant difference between the a* values of WE8 and WE10. This treatment reduced yellow tones in enriched chocolates where the b* values of the samples WE6 and WE8 did not differ from each other	–	Lončarević et al. (2018)
White chocolate	Encapsulated green tea extract (GTE) (60, 80, and 100 g/kg)	Encapsulated	Fortification of chocolate led to an increase in the volume‐weighted mean from 15.43 μm in white chocolate to 19.34 μm in chocolate with the maximum concentration of GTE. Also, the Casson viscosity and yield stress of fortified samples increased, reaching 1.24 Pas and 8.19 Pa, respectively, in the sample with the maximum amount of GTE	Fortification reduced the gloss intensity on the chocolate surface compared to the white chocolate surface, but there was no difference between the values of gloss obtained for enriched chocolates Fortification could not affect the hardness and melting of fortified chocolates. Still, a sandy texture was developed while consuming the enriched chocolates, mainly in the sample with the maximum dose of GTE. Also, there was a reduction in the samples' cocoa butter flavor and sweetness based on added concentrations	The surface color of enriched chocolates changed according to the fortification dose, where all fortified samples after 12 months of storage had a slightly lighter color. The values for a* in fortified samples are also located near the cross‐section of the coordinates a* and b* (0.77 for GTE60, 1.00 for GTE80, and 1.28 for GTE100), and the values were dramatically different from each other	Fortification with GTE enhanced the antioxidant capacity (mmol Trolox equivalents/kg) from 1.22 in white chocolate to 16.12. Antioxidant capacity dipped to 44.14% after 12 months of storage	Lončarević et al. (2019)
Dark chocolate	Oleanolic and ursolic acids isolated from *Mansoa hirsuta* DC (0.06%)	Free	The fortified sample obtained a 20 μM particle size.	In the hedonic scale's acceptance region, the fortified chocolate had a mean score of 7.29 ± 1.43, and the fortified samples were appropriately accepted	–	–	Milagres et al. (2019)
Milk chocolate	Cinnamon essential oil (0.1%, 0.3%, and 0.5%)	Free	–	It was concluded that fortification of chocolate with 0.3% and 0.5% of cinnamon essential oils resulted in too much spicy taste perception leading to less consumer acceptance. The results revealed that milk chocolate with the lowest fortification amount was the most preferred sample	Fortified samples with 0.1% and 0.3% of cinnamon essential oil were in the range of red color. While the sample with the dose of 0.5% cinnamon essential oil was in the range of red‐yellow color. Hence, it can be concluded that the color was getting brighter by enhancing the dosage of fortification	–	Ilmi et al. (2017)
Chocolate rice crispy bar	Encapsulated freeze‐dried *Scenedesmus obliquus* (5% w/w)	Encapsulated		Three panelists could detect the fishy odor from the fortified sample, and they preferred the flavor of the sample without fortification. Furthermore, no significant difference was seen in the texture of both samples as half of them preferred both samples			Hlaing et al. (2020)

## 8 CONCLUSION AND FUTURE PROSPECTS

As it was observed, in terms of the high volume of chocolate consumption worldwide and the special attention of people of all ages to its consumption, it is possible to achieve a health‐oriented and popular product. On the other hand, we should not dismiss any potential negative effects that fortifying active substances may have on the desirable qualities of chocolate. Different fortification methods should be developed and optimized. Considering other quality defects in chocolate that could arise from defective production stages or inappropriate fortification methods, it is possible to produce a health‐promoting product with higher functional, nutritional, and healing properties.

Among the plant substances, the substances with rich polyphenol content, such as apple and olive, had a higher effect on treating and reducing the effects of obesity, overweight, hypertension, stress, cardiovascular failure, congestive heart failure, and diabetes. Finally, it seems that chocolate fortification could be done with other health‐promoting substances such as probiotic bacteria, polyunsaturated fatty acids (PUFA), vitamins, minerals, substances of oilseeds, natural colors, algae, and other plant items in chocolate to evaluate their therapeutic role in disease treatment. Even though the fortification of chocolate has been shown to have positive effects, it is important to consider the researchers' worries about long‐term or excessive chocolate consumption or when there is no effective concentration of plant‐based substances used in the chocolate‐making process.

## AUTHOR CONTRIBUTIONS

**Amirhossein Abedini:** Writing – original draft (equal). **Samira Dakhili:** Writing – original draft (equal). **Sara Bazzaz:** Writing – original draft (equal). **Saba Kamaladdin Moghaddam:** Writing – original draft (equal). **Maryam Mahmoudzadeh:** Conceptualization (equal); project administration (equal). **Hashem Andishmand:** Supervision (equal); writing – review and editing (equal).

## CONFLICT OF INTEREST STATEMENT

There are no conflicts of interest to declare.

## ETHICS STATEMENT

This study does not involve any human or animal testing.

## Data Availability

No datasets were generated or analysed during the current study.
